# New library of phase-change materials with their selection by the Rényi entropy method

**DOI:** 10.1038/s41598-023-37701-0

**Published:** 2023-06-27

**Authors:** Vladimir Kulish, Navid Aslfattahi, Michal Schmirler, Pavel Sláma

**Affiliations:** Department of Thermodynamics & Fluid Mechanics, Faculty of Mechanical Engineering, Czech Technical University in Prague, Prague, Czech Republic

**Keywords:** Mechanical engineering, Thermodynamics

## Abstract

The secret to the successful and widespread deployment of solar energy for thermal applications is effective and affordable heat storage. The ability to provide a high energy storage density and the capacity to store heat at a constant temperature corresponding to the phase transition temperature of the heat storage material (phase-change material or PCM) make latent heat storage one of the most alluring methods of heat storage. Today, it can be challenging to obtain all the published data on PCM qualities, including relevant non-thermodynamic properties in addition to thermodynamic ones. The developed new PCM library contains various types of PCMs which possess broad range of operation temperatures. This new library consists of 500 substances along with nine associated properties such as phase change temperature, solidification temperature, maximum operation temperature, density, latent heat and specific heat capacity, thermal conductivity, cycleability and ignition temperature. Furthermore, a new PCM selection method, based on calculating the Rényi entropy for a given set of selection criteria, has been proposed. The newly developed selection method requires no subjective judgements. The idea of the method is inspired by earlier applications of fractal analysis methods in many areas of research.

## Introduction

The solar energy as the most prominent source of renewable energies is regarded as one of the most efficient and reliable technologies for supplying energy. However, further improvements in thermal energy storage systems are required to boost up the efficiency of current solar energy systems. The capability of phase change materials (PCMs) in terms of high energy storage density and the capacity to store heat at a constant temperature corresponding to the phase transition temperature plays vital role in the advancement of solar energy systems and makes latent heat storage as one of the most alluring methods of heat storage^1^.

Any latent heat thermal energy storage system must have the minimum of three of the following elements: a heat storage substance that transitions from a solid to a liquid at the desired operating temperature range, where the majority of the heat added is stored as the latent heat of fusion; a containment for the storage substance; and a heat exchanging surface for transferring heat from the heat source to the storage substance and from the latter to the heat source^2^.

Thus, knowledge of two fundamentally distinct topics—heat storage materials and heat exchangers—is required for the creation of a latent heat thermal energy storage (LHTES) system^3^.

The topic of heat-of-fusion storage materials is the exclusive focus of the present paper, whereas heat exchangers will be considered in subsequent studies.

At present, there are 38 commercial organisations associated with PCMs and 10 material selection databases and software/modelling tools are available^4^. Even with these vast resources, retrieving all desired properties of PCMs still remains challenging. This is because, among the available resources, many of them are devoted to only several PCM properties and their formats are not standardised in most of the cases^5^.

The major goal of this study is to create a library that makes it easier to choose the best PCM for a given application. Furthermore, a new selection method, based on calculating the Rényi entropy for a given set of selection criteria, has been proposed.

## Methodology

An effective way to store thermal energy is employing a latent heat storage system with organic/inorganic phase change material (PCM). PCMs can absorb and/or release a remarkable amount of latent heat as a result of a phase transition when the phase transition temperature is within a specified temperature range^6^.

Currently, heat accumulators based on phase transitions are most widely used. This method of accumulation is used in ventilation and air conditioning, heating and hot water systems, including for storing solar energy (for example, in solar systems).

Usually, PCMs can be primarily divided to be pure components such as unary systems or mixtures such as binary, ternary, quaternary and multicomponent systems. Organic PCMs could be considered as alkanes, alkenes, fatty acids, polyols, alkanols while inorganic PCMs are mainly based on salts (pure salts, salt blends and salt hydrates) and metals (pure metals or metal alloys). Multicomponent blends have gained immense attention due to their possibility for adjusting the melting temperature towards applications requirements but these blends come with inherent complexities^7^. Blends could be from organics or inorganics respectively, or even as a mixture of the two categories^3^. In mixtures (or blends), congruently melting compounds or congruently melting solid solutions are the two best composition types perfectly suited as PCM candidates. Because they are congruent melting (they behave like pure component materials). Then, eutectics (as another potentially suitable composition type as a PCM are suitable as PCMs, only if they do not supercool. However, eutectics are not congruent melting by theory. Only because they form an "intimate mix" where the total composition of the solid phases in equilibrium have the same composition of that liquid, they exhibit a congruent melting-like behaviour. However, if a eutectic supercools, the total composition of the solid phases will vary from that of the liquid, causing that eutectic to phase separate. In contrast, congruently melting compositions (either forming such a solid solution or a compound) will not phase separate even if they supercool, as they are even theoretically congruently melting. Where, the liquid and the solid phase in equilibrium at that composition always (and truly) have the same composition. All incongruently melting compositions (including peritectics which form incongruently melting compounds) in any blend is unsuitable as a PCM because they will always phase separate (and sometimes will definitely supercool, such as peritectics).

**Figure 1:**
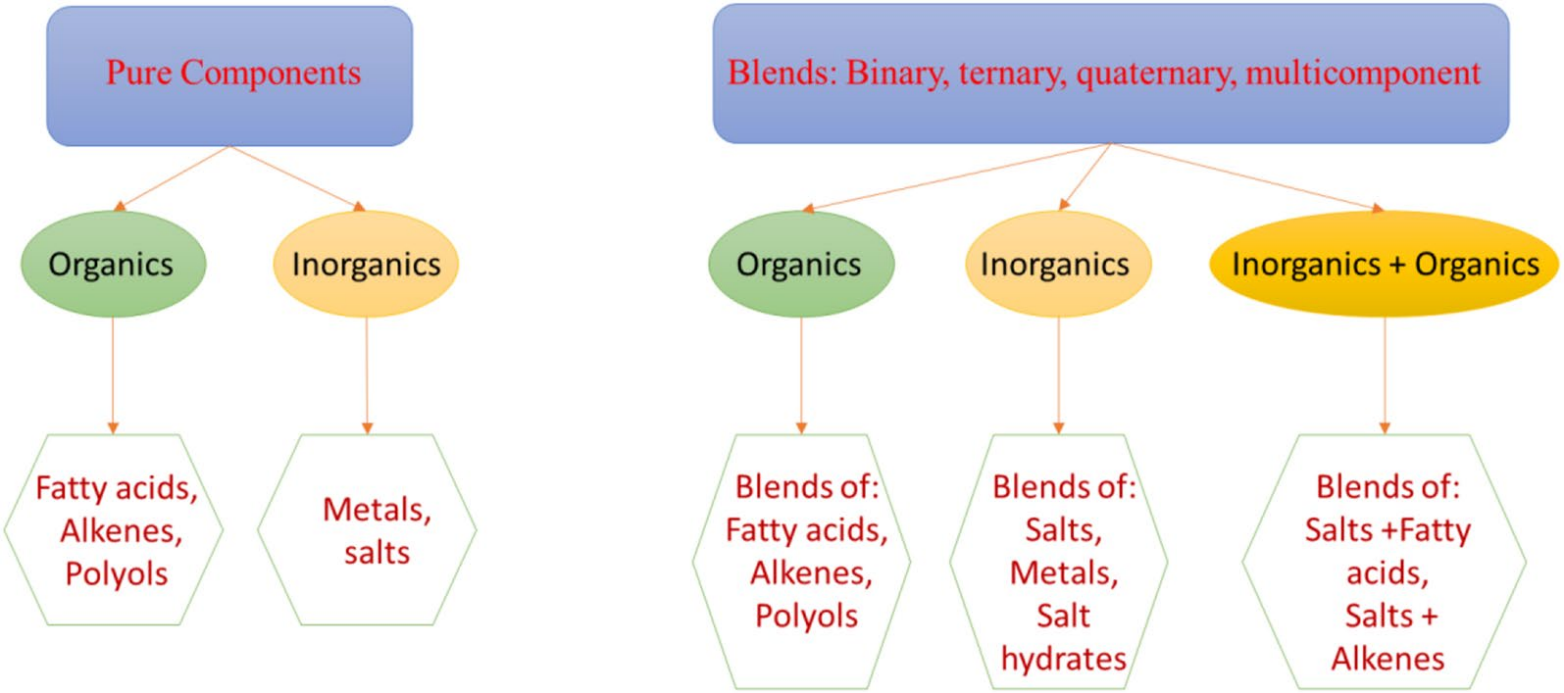
Families of PCMs used for heat storage.

Figure 1 shows families of PCMs used for heat storage.

Some features of organic PCMs can be summarised as follows:

Paraffin PCMs typically have a relatively sharp enthalpy profile with well-defined melting and crystallisation temperatures. They have a low vapour pressure but are relatively expensive and incompatible with plastic containers. The average thermal conductivity of paraffins is about 0.2 W/(m·°C), and the heat of phase transition in most cases varies from 150 to 270 kJ/kg^8^.

Among non-paraffins, fatty acids are the most popular. The latent heat of many fatty acids is between 150 and 220 kJ/kg, and the environmental and fire safety is much higher than that of paraffin hydrocarbons. The average thermal conductivity of acids varies in the range from 0.15 to 0.17 W/(m °C)^9^.

Some features of inorganic PCMs can be summarised as follows:

In the group of inorganic materials, only hydrates of salts are currently widely used. Hydrated salts are composed of inorganic salts and one or more water molecules^10^. In the process of phase transformation, hydration or dehydration of salts occurs. When dehydrated, the salt hydrate can be converted to an anhydrous form with complete separation of salt and water, or to a salt hydrate with less water (This happens only if the chosen salt hydrate is an incongruently melting compounds, such as a peritectic)^11^. The average latent heat of salt hydrates is quite high, about 250 kJ/kg, and their average thermal conductivity is about 0.5 W/(m·°C), which is higher than that of organic PCM^12^. Salt hydrates are available in large quantities, are low cost, non-flammable, and have a relatively high latent heat per unit volume. At the same time, they are characterised by moderate to high corrosiveness concerning metals and a noticeable change in volume during melting.

For incongruently melting salt hydrates with incongruent melting during dehydration, the density of the anhydrous salt can cause it to sink to the bottom of the container^13^. When the crystallization temperature is reached, the salt will be deposited at the bottom, and some of it will not be able to reabsorb the water. The total volume of salt involved in the phase change will decrease, with which the effectiveness of the PCM will also decrease.

Salt hydrates are compatible with plastics, which makes their packaging easier and cheaper.

Compared to organic PCMs, inorganic materials generally have similar mass-specific enthalpies but higher volume-specific enthalpies due to their high density.

Some features of eutectic PCMs can be summarised as follows:

Of the organic eutectics, mixtures of fatty acids are the most frequently studied. Many combinations of inorganic eutectic mixtures have yet to be tested and validated.

Eutectics are characterised by the possibility of obtaining an accurate phase transition temperature and high cost.

Desired properties of PCMs can be divided into four classes as follows^2^:

I. Thermodynamic properties
  1. A melting point in the desired operating temperature range;
  2. High latent heat of fusion per unit mass (a lesser amount of PCMs stores a given amount of energy);
  3. High density (a smaller container volume holds PCMs);
  4. High specific heat (for additional sensible heat storage effects);
  5. High thermal conductivity (small temperature gradients during charging and recharging PCMs);
  6. Congruent melting;
  7. Small volume changes during phase transition (simple containment and heat exchanger geometry can be used);
II. Kinetic properties.
  8. Little or no supercooling during freezing (the melt should crystallise at its thermodynamic freezing point);
III. Chemical properties.
  9. Chemical stability;
  10. No chemical decomposition (high system life is assured);
  11. Non-corrosiveness to construction materials;
  12. Non-poisonous, non-flammable, non-explosive;
IV. Economic properties.
  13. Available in large quantities;
  14. Inexpensive.

The proposed new PCM library contains various types of organic/inorganic PCMs which possess broad range of operation temperatures. This new library consists of almost 500 substances along with nine associated properties as the most important parameters related to these family of materials. The aforementioned nine associated properties include phase change temperature, solidification temperature, maximum operation temperature, density, latent heat and specific heat capacity, thermal conductivity, cycleability and ignition temperature. According to the collected data, the highest latest heat capacity among the 500 substances belongs to H725-PlusICE which is 602 kJ/kg. PCM-HS01P has exposed the highest thermal conductivity value among the 500 PCM (2.5 W/mK), while the acquired results for thermal conductivity results cover broad range from 0.132 to 2.5 W/mK.

There should be noted that the most available data related to these family of materials have been extracted through all valid available databases.

## New PCM library

To develop the PCM library presented in this paper, all of the available resources including Journal articles, books, commercial suppliers and relevant websites were checked with most of the PCM manufacturers contacted in person. As a result, more than 500 substances were introduced in the library taking in consideration not only the thermodynamic properties but also other desired PCM properties (e.g., corrosiveness) where available.

Table 1 presents a representative sample of the new PCM library, in which only the most important thermodynamic properties are listed. In particular, where available, the ranges of melting and solidification temperatures are provided. The latter features should enable engineers and researchers to take into account the phase transition with hysteresis.

**Table 1:** Representative sample of the new PCM library.

	PCM	Type	Phase change temperature (°C)	Solidification temperature (°C)	Maximum operation temperature (°C)	Density (kg/m^3^)	Latent heat capacity (kJ/kg)	Specific heat capacity (kJ/kgK)	Thermal conductivity (W/mK)	Special notes	Reference
1	E-68/PlusICE	Inorganic	− 68	− 70	100	1360	205	3.15	0.54	Copper (UNSC38600) demonstrates the greatest corrosive behavior in interaction with this PCM. Aluminium (UNS A92024) exhibits the least corrosion rate in interaction with this PCM	https://www.pcmproducts.net/files/PlusICE%20Range%202021-1.pdf
2	A2-PlusICE	Organic	2	0	150	765	230	2.2	0.21	Weak interaction with the metal surface by van der Waals interactions and can be removed with heat and/or shear possibly resulting in a localized attack on the steel surface	https://www.pcmproducts.net/files/PlusICE%20Range%202021-1.pdf
3	n-Tetradecane (Paraffin 14-Carbons)	Organic	5.5	1.5	55	771	228	2.19	0.12	Weak interaction with the metal surface by van der Waals interactions and can be removed with heat and/or shear possibly resulting in a localized attack on the steel surface	10.1016/j.est.2017.11.005
4	S10-PlusICE	Inorganic	10	7	60	1470	170	1.9	0.43	AA 6061 aluminium exposes good corrosion stability. AISI 1050 carbon steel and CW024A copper alloys show significant electrochemical activity, together with a large amount of corrosion products on the surface	https://www.pcmproducts.net/files/PlusICE%20Range%202021-1.pdf
5	Isopropyl palmitate	Organic	11	8.5	125	852	98	1.78	0.24	Stainless steel alloys and aluminium alloys show good compatibility with these types of PCMs	10.1016/j.est.2017.11.005
6	Paraffin microcapsules (RT15)	Organic	15	12	280	928	49.3	20	0.5	Stainless steel alloys and aluminium alloys show good compatibility with these types of PCMs	https://www.rubitherm.eu/
7	Caprylic acid	Organic	16.3	14	80	901	148	2.06	0.15	Stainless steel alloys and aluminium alloys show good compatibility with these types of PCMs	10.1016/j.est.2017.11.005
8	Dodecanol	Organic	17.5	14	125	831	202.6	2.44	0.14	Weak interaction with the metal surface by van der Waals interactions and can be removed with heat and/or shear possibly resulting in a localized attack on the steel surface	10.1016/j.matchemphys.2022.126067
9	Butyl stearate	Organic	19	16.5	238	861	140	0.46	0.14	Stainless steel alloys and aluminium alloys show good compatibility with these types of PCMs	10.1016/j.est.2017.11.005
10	KF.4H_2_O	Inorganic	18.5	16.5	120	1437	231	1.84	0.48	Aluminium and steel should be avoided in this kind of PCM containers due to their high corrosion rates. Slower corrosion has been observed for brass and copper. Stainless steel has been evaluated as corrosion resistant	10.1016/j.est.2017.11.005
11	CaBr_2_.6H_2_O	Inorganic	34	32	96	2194	115.5	0.37	0.57	Aluminium and steel should be avoided in this kind of PCM containers due to their high corrosion rates. Slower corrosion has been observed for brass and copper. Stainless steel has been evaluated as corrosion resistant	10.1016/j.est.2017.11.005
12	X40-PlusICE	Inorganic	40	37	125	1046	150	1.67	0.36	Weak interaction with the metal surface by van der Waals interactions and can be removed with heat and/or shear possibly resulting in a localized attack on the steel surface	https://www.pcmproducts.net/files/PlusICE%20Range%202021-1.pdf
13	Myristyl alcohol-C_14_H_30_O	Organic	40.62	38.5	152	824	202.75	2.4	0.942	Stainless steel alloys and aluminium alloys show good compatibility with these tupe of PCMs	10.1002/est2.80
14	RT42 Wax	Organic	45	42	72	880	139.4	3.5	0.2	Stainless steel alloys and aluminium alloys show good compatibility with these tupe of PCMs	https://www.rubitherm.eu/
15	S48-PlusICE	Inorganic	48	46	120	1565	110	2.35	0.61	AA 6061 aluminium exposes good corrosion stability. AISI 1050 carbon steel and CW024A copper alloys show significant electrochemical activity, together with a large amount of corrosion products on the surface	https://www.pcmproducts.net/files/PlusICE%20Range%202021-1.pdf
16	Sodium Thiosulfate Pentahydrate	Inorganic	48.3	47	100	1740	210.74	0.2	0.77	Aluminium and steel should be avoided in this kind of PCM containers due to their high corrosion rates. Slower corrosion has been observed for brass and copper. Stainless steel has been evaluated as corrosion resistant	10.1016/j.est.2017.11.005
17	S50-PlusICE	Inorganic	50	48	120	1545	110	2.41	0.62	AA 6061 aluminium exposes good corrosion stability. AISI 1050 carbon steel and CW024A copper alloys show significant electrochemical activity, together with a large amount of corrosion products on the surface	https://www.pcmproducts.net/files/PlusICE%20Range%202021-1.pdf
18	CH_3_COONa.3H_2_O	Inorganic	58	56	380	1450	265	0.97	0.7	Aluminium and steel should be avoided in this kind of PCM containers due to their high corrosion rates. Slower corrosion has been observed for brass and copper. Stainless steel has been evaluated as corrosion resistant	10.1016/j.est.2017.11.005
19	n-Dotriacontane (Paraffin 32-Carbons)	Organic	69.5	67.5	158.4	800	170	3.06	0.154	Stainless steel alloys and aluminium alloys show good compatibility with these types of PCMs	10.1016/j.est.2017.11.005
20	RT 70HC	Organic	70	68	100	880	260	2	0.2	Strong corrosion effect on metalls	https://www.rubitherm.eu/
21	RT80HC	Organic	78	79	110	900	220	2	0.2	Strong corrosion effect on metalls	https://www.rubitherm.eu/
22	A78-PlusICE	Organic	78	75	250	890	225	2.22	0.23	Week interaction with the metal surface by van der Waals interactions and can be removed with heat and/or shear possibly resulting in a localized attack on the steel surface	https://www.pcmproducts.net/files/PlusICE%20Range%202021-1.pdf
23	X80-PlusICE	Inorganic	80	78	190	1193	160	1.52	0.36	Copper and carbon steel show signs of surface corrosion and change of color for long-term exposition to these type of PCMs	https://www.pcmproducts.net/files/PlusICE%20Range%202021-1.pdf
24	RT 82	Organic	82	80	100	880	170	2	0.2	Strong corrosion effect on metalls	https://www.rubitherm.eu/
25	X90-PlusICE	Inorganic	90	88	190	1200	170	1.51	0.36	Copper and carbon steel show signs of surface corrosion and change of color for long-term exposition to these types of PCMs. Moreover, they both achieve higher CR values and more pronounced mass loss compared to Aluminium and Brass. Aluminium and Stainless Steel are the best suited for the role of PCM container	https://www.pcmproducts.net/files/PlusICE%20Range%202021-1.pdf
26	LiNO_3_	Inorganic	253.8	249	485	2380	360.8	1.62	0.59	Aluminium and steel should be avoided in this kind of PCM containers due to their high corrosion rates. Slower corrosion has been observed for brass and copper. Stainless steel has been evaluated as corrosion resistant	10.1016/j.renene.2021.03.061
27	H300-PlusICE	Inorganic	302	298	500	1900	130	1.55	0.54	Copper and carbon steel show signs of surface corrosion and change of color for long-term exposition to these types of PCMs. Moreover, they both achieve higher CR values and more pronounced mass loss compared to Aluminium and Brass. Aluminium and Stainless Steel are the best suited for the role of PCM container	https://www.pcmproducts.net/files/PlusICE%20Range%202021-1.pdf
28	H700-PlusICE	Inorganic	699	687	800	2410	250	1.6	0.57	Copper and carbon steel show signs of surface corrosion and change of color for long-term exposition to these types of PCMs. Moreover, they both achieve higher CR values and more pronounced mass loss compared to Aluminium and Brass. Aluminium and Stainless Steel are the best suited for the role of PCM container	https://www.pcmproducts.net/files/PlusICE%20Range%202021-1.pdf

Table 1 contains various types of organic/inorganic PCMs which possess broad range of operation temperatures. This Table demonstrates representatives of almost 500 substances along with nine associated properties as the most important parameters related to these family of materials. In addition, the main Table contains two more parameters including cyclability and ignition temperature, however, these two parameters are available rarely (almost 10% of substances) which has caused the prevention of including these two parameters within the representative Table. There should be noted that the most available data related to these family of materials have been extracted through all valid available databases.

Twenty-eight PCMs out of more than five hundred are included into the representative sample. They are chosen so as to cover the entire range of the PCM operation temperatures as the developed new library contains various types of PCMs with different operation temperature. In contrast with the complete library, the representative sample does not include cyclability and the ignition temperature among the PCM properties. This is because the said two properties are rarely available based on the careful observation conducted by the authors (in about ten per cent of cases only).

## Selection method based on the Rényi entropy

Even with an available comprehensive library of PCM properties, selecting a proper PCM or several suitable PCMs for a given application is often a challenge. Many existing PCM databases merely provide data but no selection tools. Some databases require sophisticated selection tools, which most often are not free of charge^13^.

As regards selection methods, many of them require a subjective set of selection criteria. Thus, for instance, to apply the analytic hierarchy process for PCM selection, one needs to construct a selection matrix based on guesswork, that is, the relative importance of selection criteria is set by a human operator by assigning values from 0 to 9 and, in addition, taking into account even such parameters as the PCM price and availability on the market^14^.

In this study, a new selection method is proposed, which requires no subjective judgements, i.e., the method uses PCM physical properties given by numbers but not the ones described by words. The idea of the method is inspired by earlier applications of fractal analysis methods in many areas of research ranging from applied mathematics (especially chaos theory)^15^ to physics^16,17^, to image and signal processing (especially in bio-medical applications)^18,19^.

It is worth noting at this point that, from all the PCMs listed in the database discussed above, the selection method presented in this Section automatically pre-selects several (usually four-five) PCMs based solely on numerical values of their physical properties. Hence, the method does not use parameters given in a descriptive way (e.g., type of PCM, its composition, corrosivity, etc.). However, as can be seen from the following example, the method renders good pre-selection results, which allow one to significantly narrow choices and reduce time needed for further manual processing of thus pre-selected PCMs (e.g., to distinguish between pure components and blends, congruently and incongruently melting compositions, etc.).

A detailed description of the selection method now follows:

For a given discrete probability distribution, ${p}_{1},{p}_{2},...,{p}_{n}$, the Rényi entropy of order $\alpha$ ($-\infty <\alpha <+\infty$) is defined as^20^

1 $${H}_{\alpha }=\frac{1}{1-\alpha }\mathrm{log}\left(\sum_{i=0}^{n}{p}_{i}^{\alpha }\right),$$

where the logarithm is conventionally taken to be base 2, especially in the context of information theory where bits are used.

As *α* approaches zero, the Rényi entropy increasingly weighs all events with nonzero probability more equally, regardless of their probabilities. In the limit for *α* → 0, the Rényi entropy is just the logarithm of *n*. The limit for *α* → 1 is the Shannon entropy^21^.

2 $${H}_{1}=-\stackrel{n}{\sum_{i=0}}{p}_{i}\,\,\mathrm{log}\,\,{ p}_{i}$$

As *α* approaches infinity, the Rényi entropy is increasingly determined by the events of highest probability. Conversely, as *α* approaches minus infinity, the Rényi entropy is increasingly determined by the events of lowest probability.

${H}_{\alpha }$ is non-increasing in α for any given probability distribution ${p}_{1},{p}_{2},...,{p}_{n}$. The latter can be easily proven by differentiation, as

3 $$-\frac{d{H}_{\alpha }}{d\alpha }=\frac{1}{(1-\alpha {)}^{2}}\mathrm{log}\left[\stackrel{n}{\sum_{i=0}}{z}_{i}\mathrm{log}\left(\frac{{z}_{i}}{{p}_{i}}\right)\right],$$

where ${z}_{i}={p}_{i}^{\alpha }/\stackrel{n}{\sum_{j=1}}{p}_{j}^{\alpha }$ and which is proportional to the Kullback–Leibler divergence (always non-negative)^22^. Thus, ${H}_{{\alpha }_{1}}\ge {H}_{{\alpha }_{2}}$ for ${\alpha }_{1}<{\alpha }_{2}$. For instance, ${H}_{-\infty }\ge {H}_{0}\ge {H}_{+\infty }$, which, in general, makes $H(\alpha )$ a Z-shaped function.

The fact that the set of the Rényi entropies includes the Shannon entropy and is closely related to the Kullback–Leibler divergence makes it a powerful tool for a selection process, for which no initial subjective guessing is needed.

Indeed, if the task is to select a PCM based on *m* selection criteria, the corresponding probabilities, ${p}_{1},{p}_{2},...,{p}_{m}$, can be defined as ${p}_{i}={q}_{i}/{(mq}_{0,i})$ for ${q}_{0,i}>{q}_{i}$ and ${p}_{i}={(q}_{0,i}/m{q}_{i})$ for ${q}_{i}>{q}_{0,i}$, where ${q}_{0,i}$ denotes the desired value of the selection criteria, while ${q}_{i}$ stands for the value available in the library. Notice that division by *m* renders the normalisation condition in case of an exact match with the reference quantities, that is, ${p}_{0,i}=1/m$ for all *i*.

The above definition of “probabilities” renders a simple selection algorithm:

1. For all PCMs within the library, calculate ${p}_{1},{p}_{2},...,{p}_{m}$ based on the desired values ${q}_{0,i}$.
2. Calculate the complementary probabilities as ${p}_{c}=1-\sum_{i=1}^{m}{p}_{i}$ for all PCMs to achieve the completeness of all probability distributions.
3. For all PCMs within the library, compute $H(\alpha )$ using Eq. (1) for $\alpha \ne 1$ and Eq. (2) for $\alpha =1$. Notice that for most practical purposes the range $-50\le \alpha \le 50$ or even narrower is sufficient.
4. Choose several (usually no more than five) PCM candidates, for which ${H}_{1}$ are the least (${H}_{-\infty }={H}_{1}={H}_{+\infty }=\mathrm{log}m$ in case of an exact match [see Eq. (2)]).
5. Rank the chosen PCMs in accord with the flatness of their $H(\alpha )$ functions, that is, the candidate with the least value of ${H}_{-\infty }-{H}_{+\infty }$ is the best.

To illustrate how the proposed selection method works, consider the case, which arose in the course of designing a real latent heat accumulator based on demand from an industry partner. During laboratory tests, it has been found that PCM RT80HC, which was preselected by the customer as the best candidate, showed high corrosivity under required operation conditions. In view of this, several other PCMs were to be proposed as potential candidates.

Desired values of the selection criteria, ${q}_{0,i}$, which are the physical properties of RT80HC, are summarised in Table 2.

**Table 2:** Desired values of the selection criteria (physical properties of RT80HC).

Property	${T}_{ph}$, °C	${T}_{s},$°C	${T}_{\mathrm{max}}$, °C	$\rho ,$ kg/m^3^	${h}_{f},$ kJ/kg	${c}_{p},$ kJ/(kg K)	$k$, W/(m K)
*i*	1	2	3	4	5	6	7
${q}_{0,i}$	78	79	110	900	220	2	0.2

In Table 2, the following nomenclature is used: ${T}_{ph}$—phase-change temperature; ${T}_{s}$—solidification temperature; ${T}_{\mathrm{max}}$—maximum operation temperature; $\rho$—density; ${h}_{f}$—latent heat capacity; ${c}_{p}$—specific heat capacity; $k$ – thermal conductivity.

A special remark is to be made about assigning values to those ${q}_{0,i}$, which represent qualitative criteria such as, for instance, PCM type (e.g., organic/inorganic), corrosivity, etc. In principle, for some characteristics, the latter can be done by assigning binary values (e.g., organic = 1/inorganic = 0, or vice versa), while, for characteristics, whose values cannot be expressed in terms of binary values (e.g., corrosivity), other than the binary system can be used (e.g., absent = 0, low = 1, medium = 2, high = 3). However, the latter may bring an element of subjectivity into the proposed method of choosing PCMs. To avoid this, it is proposed—especially for the database in question—to limit properties used to calculate the probabilities to those, which can be given as numerical values.

Indeed, the latter limitation causes no problem at all, because, from four-five PCMs selected based on their $H(\alpha )$ values, it is very easy to choose the best by simple inspection. Moreover, in some cases (e.g., binary characteristics such as organic/inorganic), there is no need to calculate $H(\alpha )$ values for all PCMs in the database, but only for PCMs, which belong to a desired type.

It is obvious that the accuracy of the probability values as well as of the $H(\alpha )$ values is the same as the accuracy with which the values of physical properties are given in the PCM database—three significant digits in the present case. Moreover, while computing the values of ${H}_{\pm \infty }$, changing ${\alpha }_{\mathrm{min}/\mathrm{max}}$ from $\pm 50$ to $\pm 100$ affects the result by 1% only. Thus, ${\left|\alpha \right|}_{\mathrm{max}}=50$ in this study.

A special remark is to be made about the presence of the complementary probability values, ${p}_{c}$, in Table 3. These values are calculated simply as ${p}_{c}=1-\sum_{i=1}^{m}{p}_{i}$ and are needed to satisfy the normalisation requirement, that is, achieve completeness of the probability distributions for PCMs to be selected.

**Table 3:** PCMs selected from the entire library by the Rényi entropy method.

PCM	${p}_{1}$	${p}_{2}$	${p}_{3}$	${p}_{4}$	${p}_{5}$	${p}_{6}$	${p}_{7}$	${p}_{c}$	${H}_{1}$	${H}_{-\infty }$	${H}_{+\infty }$	$\Delta H$
RT80HC	0.143	0.143	0.143	0.143	0.143	0.143	0.143	0	2.807	2.807	2.807	0
A78-PlusICE	0.143	0.143	0.063	0.141	0.140	0.129	0.124	0.117	2.966	3.910	2.832	1.078
X80-PlusICE	0.139	0.141	0.083	0.108	0.104	0.109	0.079	0.237	2.909	3.593	2.119	1.474
RT70HC	0.128	0.123	0.130	0.140	0.121	0.143	0.143	0.072	2.976	3.721	2.838	**0.883**
RT82	0.136	0.141	0.130	0.140	0.110	0.143	0.143	0.057	2.958	4.052	2.832	1.220

The best PCM choice is shown in bold.

As can be seen from Table 3, RT70HC has the flattest *H*-function and is to be selected to replace RT80HC.

To conclude this section, an important remark is to be made. It seems tempting to use the Shannon entropy only, to select a PCM with a desired set of properties. Such a simplistic approach, however, may lead to a situation, in which one of the properties outweighs the contributions of others. On the other hand, using the entire functions $H(\alpha )$ makes it certain that all members of the property set contribute in a more uniform way. To further support the latter claim, notice from Table 3 that X80-PlusICE has the value of ${H}_{1}$ nearest to the one of RT80HC. Yet X80-PlusICE is the worst among the preselected candidates, because it has the largest range ${H}_{-\infty }-{H}_{+\infty }$.

## Conclusions

In this study is presented a new library (database) to help during the selection process of PCM. Using resources of 38 commercial organisations associated with PCMs and 10 material selection databases, more than 500 substances were introduced in the library taking in consideration not only the thermodynamic properties but also other desired PCM properties (e.g., corrosiveness) where available.

There is no need to say that the presented PCM library is not perfect and further improvements are planned.

In addition, to select a PCM from the library, a new selection method based on calculating the Rényi entropy has been proposed. The main advantage of the method is that it does not require any prior subjective assumptions about the importance of PCM properties, which contribute into the selection process. The said selection method has been tested in the course of designing a real heat accumulator to replace a PCM, which showed high corrosiveness under required operation conditions.

## Data Availability

The PCM library, generated during this study, is available for purchase under various subscription plans at https://fluids.fs.cvut.cz/licensing/pcm-catalog/.
